# Early Paradoxical Increase of Dopamine: A Neurochemical Study of Olfactory Bulb in Asymptomatic and Symptomatic MPTP Treated Monkeys

**DOI:** 10.3389/fnana.2017.00046

**Published:** 2017-05-29

**Authors:** Christian Pifl, Harald Reither, Natalia Lopez-Gonzalez del Rey, Carmen Cavada, Jose A. Obeso, Javier Blesa

**Affiliations:** ^1^Center for Brain Research, Medical University of ViennaVienna, Austria; ^2^HM CINAC, Hospital Universitario HM Puerta del SurMostoles, Spain; ^3^Centro de Investigacion Biomedica en Red sobre Enfermedades Neurodegenerativas (CIBERNED), Instituto Carlos III, Ministerio de Ciencia e InnovacionMadrid, Spain; ^4^Departamento de Anatomia, Histologia y Neurociencia, Facultad de Medicina, Universidad Autonoma de MadridMadrid, Spain

**Keywords:** olfactory bulb, MPTP, dopamine, noradrenaline, serotonin, amino acid neurotransmitters

## Abstract

Parkinson’s disease (PD) is a neurodegenerative disease with both motor and non-motor manifestations. Hyposmia is one of the early non-motor symptoms, which can precede motor symptoms by several years. The relationship between hyposmia and PD remains elusive. Olfactory bulb (OB) pathology shows an increased number of olfactory dopaminergic cells, protein aggregates and dysfunction of neurotransmitter systems. In this study we examined tissue levels of dopamine (DA) and serotonin (5-hydroxytryptamine, 5-HT) and their metabolites, of noradrenaline (NA) and of the amino acid neurotransmitters aspartate, glutamate, taurine and γ-aminobutyric acid in OBs of 1-methyl-4-phenyl-1,2,3,6-tetrahydropyridine (MPTP) treated *Macaca fascicularis* in different stages, including monkeys who were always asymptomatic, monkeys who recovered from mild parkinsonian signs, and monkeys with stable moderate or severe parkinsonism. DA was increased compared to controls, while neither NA and 5-HT nor the amino acid neurotransmitters were significantly changed. Furthermore, DA increased before stable motor deficits appear with +51% in asymptomatic and +96% in recovered monkeys. Unchanged DA metabolites suggest a special metabolic profile of the newly formed DA neurons. Significant correlation of homovanillic acid (HVA) with taurine single values within the four MPTP groups and of aspartate with taurine within the asymptomatic and recovered MPTP groups, but not within the controls suggest interactions in the OB between taurine and the DA system and taurine and the excitatory neurotransmitter triggered by MPTP. This first investigation of OB in various stages after MPTP administration suggests that the DA increase seems to be an early phenomenon, not requiring profound nigrostriatal neurodegeneration or PD symptoms.

## Introduction

The olfactory bulb (OB) is a neuronal structure of the forebrain being the most rostral part of the adult brain in rodents, but situated on the inferior side of the forebrain in primates. According to the prosomeric model of the brain holding that the brain is formed by an uninterrupted series of transverse subunits of the neural tube, it is dorsal of the prospective anterior commissure which is the most rostral point of the neural tube (Puelles and Rubenstein, 2015). It functions as a processor of odor information by forming a sophisticated network of multiple types of neurons transmitting the information to the olfactory cortex. The monoamine neurotransmitters dopamine (DA), noradrenaline (NA) and serotonin (5-hydroxytryptamine, 5-HT) have been shown to influence odor processing. DA is the neurotransmitter of an intrinsic subpopulation of neurons within the glomerular cell layer and affects odor discrimination learning in rats (Escanilla et al., 2009). Recently, a direct axonal dopaminergic projection from the substantia nigra (SN) to the core of the OB was described and suggested to facilitate the perception of odorants (Höglinger et al., 2015). Noradrenergic fibers from the locus coeruleus and serotoninergic fibers from the raphe nucleus project to all layers of the OB (McLean and Shipley, 1987, 1991; Gómez et al., 2005). NA modulates odor habituation (Guerin et al., 2008) and 5-HT regulates odor inputs in the OB (Petzold et al., 2009). For all monoamines effects on synaptic transmission in the OB were also shown (Jiang et al., 1996; Berkowicz and Trombley, 2000; Petzold et al., 2009).

Importantly, olfactory dysfunction is a frequent non-motor symptom of Parkinson’s disease (PD). There is structural pathology observed in the OB of individuals with PD. Lewy body pathology was also detected in the OB in patients with incidental Lewy body disease and PD (Attems et al., 2014; Saito et al., 2016). The OB volume was found reduced in several studies (Wang et al., 2011; Brodoehl et al., 2012; Li et al., 2016), whereas the number of DA neurons was increased in some reports (Huisman et al., 2004, 2008; Mundiñano et al., 2011). Dopaminergic neurogenesis also increased in OB following i.p. 1-methyl-4-phenyl-1,2,3,6-tetrahydropyridine (MPTP) intoxication in mice (Yamada et al., 2004) and after nigrostriatal DA depletion by 6-hydroxydopamine injection in the SN in mice and in the medial forebrain bundle in rat (Winner et al., 2006; Chiu et al., 2014; Höglinger et al., 2015). In addition, in the α-synuclein A53T transgenic mice there was a decrease of cholinergic neurons and acetylcholinesterase activity in mitral cell layer and an increase of dopaminergic neurons and tyrosine hydroxylase protein in the glomerular layer at 10 months (Zhang et al., 2015). Tyrosine hydroxylase expression was also found increased in the OBs of transgenic rats with α-synuclein mutations (Lelan et al., 2011). Thus, nigrostriatal DA depletion through different approaches appears to increase dopaminergic neurogenesis in the OB. The increased number of DA neurons could be a compensatory change for the lack of inhibitory modulation of mitral cells by cholinergic, noradrenergic and serotonergic olfactory afferents which are often reduced in neurodegenerative diseases (Mundiñano et al., 2011). Similarly, increased dopaminergic tone in the OB of PD patients could reflect a compensatory mechanism created by the early degeneration of other neurotransmitter systems and might contribute to the olfactory dysfunction exhibited by patients with neurodegenerative disorders (Bédard et al., 2002; Winner et al., 2006; Mundiñano et al., 2011).

In MPTP parkinsonian monkeys the number of olfactory dopaminergic neurons was reported to be nearly doubled as compared to controls (Belzunegui et al., 2007) and, in fact, cholinergic OB innervation was decreased in this model as well as in patients with PD (Mundiñano et al., 2013). Here, we have examined the OBs of *Macaca fascicularis* monkeys in four motor states after progressive parkinsonism induced by repeated administration of small doses of MPTP corresponding to different degrees of nigro-striatal lesion and DA depletion (Blesa et al., 2012).

## Materials and Methods

### Animals

Experiments were performed using OBs from 37 male cynomolgus monkeys (*Macaca fascicularis*, RC Hartelust, Tilburg, Netherlands, mean age 5.2 ± 0.9 years, mean weight 3.6 ± 0.65 kg). Animals were housed in primate cages, singly or in pairs, under controlled conditions of air exchange (16 l/min), humidity (50%) and light/dark cycles (8 am to 8 pm); food and water were available *ad libitum*. All procedures were performed according to the European Council Directive 86/609/EEC and in accordance with the Society for Neuroscience Policy on the Use of Animals in Neuroscience Research. The Ethics Committee for Animal Testing of the University of Navarra approved the experimental design.

### Experimental Protocol

Fourteen of the 37 monkeys were used as controls. The remaining 23 monkeys were treated with MPTP hydrochloride (Sigma, St Louis, MO, USA) using a low-dose regimen (0.5 mg/kg in saline administered intravenously once every 2 weeks) to obtain partial, slowly progressing degeneration of the dopaminergic nigro-striatal system (Blesa et al., 2010, 2012). The number of doses for each animal ranged from 1 to 16, and the total dose from 0.5 mg/kg to 8 mg/kg. Such a wide range in number and total dose of MPTP was determined by individual susceptibility to the toxin, which varies considerably when a slow intoxication protocol is employed. This in turn allows for different degrees of motor deficit and nigro-striatal lesion. Motor performance was tested using the Kurlan Scale (Blesa et al., 2012). To satisfy the classification criteria, each animal had to remain stable for at least 1 month in the corresponding motor state before being classified in a given group (Taylor et al., 1997; Blesa et al., 2012). The striatal biochemical characterization of these animals was published before (Blesa et al., 2012).

### Neurochemical Analysis

Samples were ultrasonicated in 30 volumes of perchloric acid, sodium bisulfite and 3,4-dihydroxybenzylamine as internal standard (final concentration 0.1 M, 0.4 mM and 20 μg/l, respectively) and centrifuged at 16,100× *g* for 10 min. For determination of 3,4-dihydroxyphenylacetic acid (DOPAC), 5-HT, 5-hydroxyindolacetic acid (5-HIAA) and homovanillic acid (HVA) 100 μl of supernatant was injected directly into a HPLC system equipped with a LiChroCART^®^ 250-4, RP18μ, 5 μm (VWR) column and a BAS (Bioanalytical System) electrochemical detector. The mobile phase consisted of 0.1 M sodium acetate buffer, pH 4.3, containing 1 mM EDTA, 0.2 mM l-heptane sulfonic acid, 0.1% triethylamine, 0.2% tetrahydrofurane and 10% methanol. For determination of DA and NA, aliquots of the supernatant were extracted with alumina oxide and injected into a HPLC system as described previously (Pifl et al., 1991) with minor modifications (LiChroCART^®^ 250-4, RP18μ, 5 μm column, HP Programmable Electrochemical Detector 1049A, mobile phase with 9% methanol). For determination of γ-aminobutyric acid (GABA), aspartate, glutamate, taurine and glycine the supernatant was diluted with H_2_O (final dilution 1:2000). One-hundred micro liter were mixed with 50 μl of OPA-reagent (25 mg o-phthalaldehyde dissolved in a mixture of 0.5 ml methanol, 25 μl 1.meercaptoethanol and 4.5 ml 0.4 sodium borate, pH 11) and after a reaction time of 2 min, 20 μl were injected by an autosampler (AS950, Jasco). The HPLC system consisted of a RP-18 column (Chromolith-Peformance RP-18e, 100 × 4.6 mm, Merck) in a column-heater set at 25°C (BFO-04 f1, W.O. Electronics) and an aqueous mobile phase containing 0.1 M sodium phosphate buffer, pH 6.0, 0.13 mM EDTA mixed with methanol by a high-pressure gradient system at a flow-rate of 1.2 ml/min (2 pumps L-7100, Merck-Hitachi) in a step-gradient of 18% methanol for 18 min and 38% methanol for the rest of the 25 min run. Derivatized amino acids were detected by a fluorescence detector (L-7480, Merck-Hitachi) with excitation at 340 nm and emission at 440 nm.

### Statistical Analysis

Data are presented as means ± SEM. Differences in neurochemical parameters were analyzed by one way analysis of variance (ANOVA) followed by the Bonferroni *post hoc* test. Pearson correlation between the parameters were calculated for the 37 single values of all animals, the 23 single values of all MPTP monkeys, 12 single values of the *asymptomatic* or *recovered* MPTP monkeys and the 14 single values of the controls. All the analysis included in the manuscript and those included in previous related articles were blinded.

## Results

Monkeys were classified in four motor states at least 1 month after the last MPTP administration: the *asymptomatic* (*n* = 6) never displayed PD-type motor symptoms; the *recovered* (*n* = 6) who had motor symptoms with a maximum Kurlan scale of 11.3 ± 1.5, but recovered to normal behavior and were sacrificed 15.3 ± 0.4 weeks after recovery; the *mildly parkinsonian* (*n* = 6) displayed motor symptoms of a Kurlan of 16 ± 1.2 to 12.3 ± 0.6; and the *severely parkinsonian* (*n* = 5) with Kurlan values of 20 ± 0.7 to 19.5 ± 1 before sacrifice (Blesa et al., 2012). The mean values of the OB weights of the 4 motor stages and the controls varied between 23 ± 5 mg and 28 ± 2 mg and did not differ significantly.

**Figure 1:**
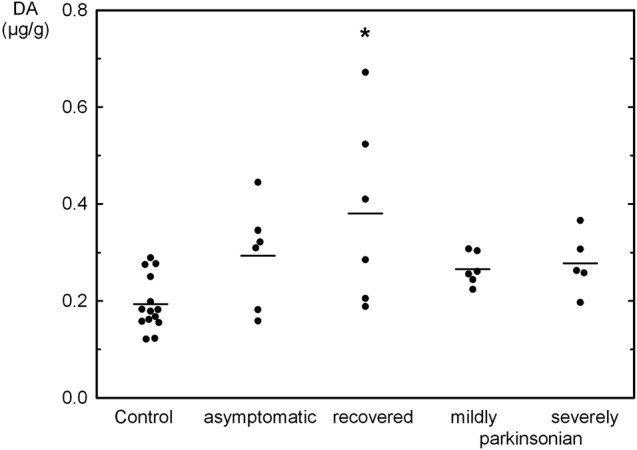
Individual values (circles) and mean values (horizontal lines) of dopamine (DA) in the olfactory bulb (OB) of *Macaca fascicularis* monkeys staying after MPTP treatment either asymptomatic (*n* = 6), recovered from parkinsonian symptoms (*n* = 6), mildly (*n* = 6) or severely parkinsonian (*n* = 5) and controls (*n* = 14). *P < 0.05 vs. control.

The tissue contents of the biogenic monoamines NA, DA and 5-HT in the OB of *controls* was quite similar ranging from 0.192 μg/g to 0.239 μg/g tissue weight. NA was not significantly changed as compared to *controls* in the various states resulting from MPTP administration with a three fold higher level in *severely parkinsonian* than in *mildly parkinsonian* monkeys (Table 1). DA levels were increased in all groups after MPTP administration (Table 1), with the highest and significant increase by 96% in the *recovered* group and gradually smaller, insignificant increases in the *asymptomatic* group (51%) and the parkinsonian group (37% and 43% in *mildly* and *severely*, respectively). Single values reveal an overlap between the recovered/asymptomatic and the control group, however at least half of the values were above the range of control values (Figure 1). 5-HT and its metabolite 5-HIAA were not significantly affected by MPTP treatment at any of the various states after administration (Table 1).

**Table 1:** Monoamine neurotransmitter parameters (μg/g_ww_) in the olfactory bulb (OB) in various stages of 1-methyl-4-phenyl-1,2,3,6-tetrahydropyridine (MPTP) parkinsonism.

Substance	Control	Asymptomatic	Recovered	Mildly parkinsonian	Severely parkinsonian
NA	0.192 ± 0.026 (14)	0.178 ± 0.031 (6)	0.157 ± 0.045 (6)	0.092 ± 0.041 (6)	0.291 ± 0.028 (5)*
DA	0.194 ± 0.015 (14)	0.294 ± 0.044 (6)	0.381 ± 0.078 (6)^#^	0.266 ± 0.014 (6)	0.278 ± 0.028 (5)
DOPAC	0.029 ± 0.002 (14)	0.032 ± 0.006 (6)	0.028 ± 0.005 (6)	0.028 ± 0.004 (6)	0.030 ± 0.003 (5)
HVA	0.306 ± 0.035 (14)	0.383 ± 0.074 (6)	0.347 ± 0.057 (6)	0.259 ± 0.026 (6)	0.365 ± 0.050 (5)
5-HT	0.239 ± 0.019 (14)	0.189 ± 0.015 (6)	0.236 ± 0.017 (6)	0.199 ± 0.024 (6)	0.327 ± 0.051 (5)
5-HIAA	0.046 ± 0.004 (14)	0.036 ± 0.008 (6)	0.036 ± 0.003 (6)	0.036 ± 0.004 (6)	0.055 ± 0.007 (5)

*p < 0.05 vs. Mildly parkinsonian, ^#^p < 0.05 vs. Control (analysis of variance, ANOVA followed by Bonferroni t-tests). Values are given as mean ± SEM (n).

Turnover of DA and 5-HT in the OB could be estimated by molar ratios calculated from single values DOPAC/DA, HVA/DA and 5-HIAA/5-HT. Neither DOPAC nor HVA values were affected by MPTP treatment, however the molar ratio DOPAC/DA was significantly reduced in the *recovered* group as compared to *control* (Table 2), whereas the DA turnover parameter HVA/DA was unchanged after MPTP administration, as well as the parameter for 5-HT turnover 5-HIAA/5-HT.

**Table 2:** Dopamine (DA) and serotonin (5-hydroxytryptamine, 5-HT) turnover (molar ratio) in the OB in various stages of MPTP parkinsonism.

Molar ratio	Control	Asymptomatic	Recovered	Mildly parkinsonian	Severely parkinsonian
DOPAC/DA	0.138 ± 0.012 (14)	0.099 ± 0.013 (6)	0.076 ± 0.013 (6)*	0.097 ± 0.013 (6)	0.104 ± 0.002 (5)
HVA/DA	1.34 ± 0.12 (14)	1.06 ± 0.12 (6)	0.90 ± 0.17 (6)	0.82 ± 0.08 (6)	1.12 ± 0.13 (5)
5-HIAA/5-HT	0.182 ± 0.013 (14)	0.170 ± 0.027 (6)	0.143 ± 0.015 (6)	0.170 ± 0.017 (6)	0.165 ± 0.023 (5)

*p < 0.05 vs. Control (ANOVA followed by Bonferroni t-tests). Values are given as mean ± SEM (n).

None of the mean values of the amino acid neurotransmitters GABA, aspartate, glutamate, taurine nor glycine was affected by MPTP significantly at any of the states after administration (Table 3). Correlation analysis of the single values between the 14 parameters all over the five experimental groups resulted in a Bonferroni-corrected significant Pearson correlation coefficient between HVA and taurine, HVA and glutamate, HVA/DA and glutamate, aspartate and taurine and between aspartate and glutamate (Table 4). None of these correlations were significant if calculated for the 14 single values in the *control* group. However, correlation analysis in the four MPTP administration groups resulted still in a significant Pearson correlation coefficient between HVA and taurine, whereas correlation analysis in the two MPTP groups without parkinsonian symptoms at the time of analysis (*asymptomatic* and *recovered* group) revealed significant correlation between aspartate and taurine.

**Table 3:** Amino acid neurotransmitters (μg/g_ww_) in the OB in various stages of MPTP parkinsonism.

Amino acid	Control	Asymptomatic	Recovered	Mildly parkinsonian	Severely parkinsonian
GABA	351 ± 20 (14)	379 ± 26 (6)	400 ± 40 (6)	379 ± 26 (6)	454 ± 13 (5)
Aspartate	230 ± 9 (14)	237 ± 18 (6)	231 ± 16 (6)	218 ± 11 (6)	232 ± 21 (5)
Glutamate	832 ± 29 (14)	757 ± 59 (6)	775 ± 39 (6)	789 ± 35 (6)	833 ± 25 (5)
Taurine	843 ± 32 (14)	881 ± 80 (6)	877 ± 52 (6)	827 ± 31 (6)	926 ± 45 (5)
Glycine	112 ± 4 (14)	99 ± 4 (6)	113 ± 13 (6)	103 ± 5 (6)	107 ± 6 (5)

*Values are given as mean ± SEM (n)*.

**Table 4:** Correlation between parameters with significant Pearson correlation coefficients after Bonferroni correction.

	All five treatment groups		All four MPTP groups		Asymptomatic and recovered MPTP groups	
	Correlation coefficient	*P*-value	Correlation coefficient	*P*-value	Correlation coefficient	*P*-value
HVA and taurine	0.663	0.00000765	0.676	0.000401	–	–
HVA and glutamate	0.590	0.000121	–	–	–	–
HVA/DA and glutamate	0.619	0.0000449	–	–	–	–
Aspartate and taurine	0.551	0.000410	–	–	0.895	0.0000823
Aspartate and glutamate	0.633	0.0000257	–	–	–	–

## Discussion

The main finding of our study is that neither the tissue content of the monoamine neurotransmitters NA, DA and 5-HT nor of their metabolites is significantly reduced at any of the various states after MPTP administration. In the same monkeys reported here striatal DA was found reduced by more than 90% (caudate in recovered group) up to 98% (caudate and putamen in the mildly and severely parkinsonian group; Blesa et al., 2012) and thalamic NA by more than 54% (ventrolateral nucleus in the parkinsonian groups) up to 70% (ventroanterior nucleus in the severely parkinsonian group; Pifl et al., 2013). In a previous study on rhesus monkeys in a group of animals assessed asymptomatic 3–5 weeks after MPTP administration widespread loss of NA and 5-HT was observed in cortical areas with a very similar dissectional and analytical approach (Pifl et al., 1991) and some of these losses are also observed in the *Macaca fascicularis* used in this study (unpublished observation). This means that noradrenergic and serotoninergic innervation of the OB by locus coeruleus and raphe nucleus, respectively, react quite differently to MPTP as compared to fibers innervating thalamic and cortical brain areas.

**Figure 2:**
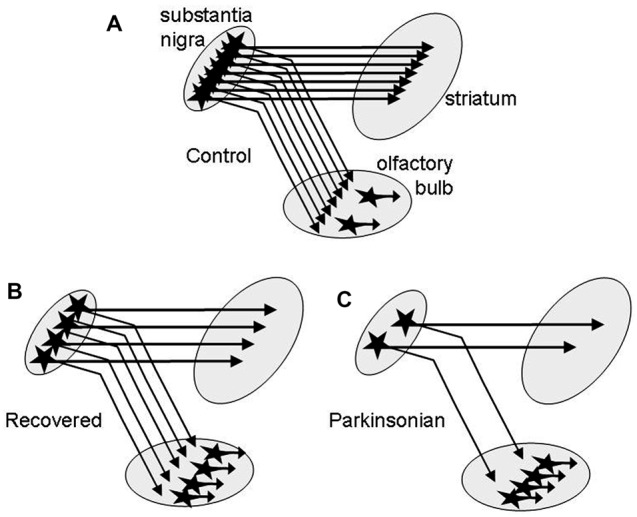
Schematic presentation of a hypothesis for the higher increase of DA in OB of recovered than in parkinsonian MPTP-treated monkeys. **(A)** DA nerve endings from the substantia nigra (SN) and intrinsic DA neurons contribute to DA tissue levels in the OB. In recovered monkeys **(B)** DA levels are increased due to more intrinsic DA neurons. In parkinsonian monkeys **(C)** DA levels are lower than in recovered due to an additional loss of nigral dopaminergic neurons innervating the OB.

The second novel finding of this study is that DA tissue content in OB is increased very early throughout all MPTP treatment groups with nearly doubling of the levels in the *recovered* group. The most obvious explanation is an increase of the number of intrinsic DA neurons in the OB reported in several studies on PD (Huisman et al., 2004; Mundiñano et al., 2011) and in animal models with nigrostriatal dopaminergic denervation by MPTP (Yamada et al., 2004; Belzunegui et al., 2007) or by 6-hydroxydopamine (Winner et al., 2006; Chiu et al., 2014). The 51% DA increase in the *asymptomatic* and the robust 96% DA increment in the *recovered* group is in keeping with neurogenesis or formation by phenotype change of DA cells in the OB in the presence of mild/moderate nigrostriatal degeneration in these monkeys (Blesa et al., 2012). Assuming that primates behave similarly to rodents, where a dopaminergic nigro-olfactory projection was described recently (Höglinger et al., 2015), the small increase of DA levels in the *mildly* and *severely* parkinsonian groups might result from a combination of increased numbers of olfactory DA cells and a more profound loss of potential nigro-olfactoral nerve terminals (Figure 2). Since all nigral dopaminergic axons innervating the rat OB appear to branch into the striatum (Höglinger et al., 2015), the lower numbers of nigral DA cells in our parkinsonian than in the *recovered* monkeys (Blesa et al., 2012) might then let expect lower DA innervation of the OB in these groups, assuming that the nigro-olfactory projection is present in macaques as well as in rats.

The increase of dopaminergic periglomerular neurons in neurodegenerative disorders was recently explained by potentially reduced noradrenergic and serotonergic inputs with a subsequent reduced inhibitory modulation of the mitral cells resulting in hyperactivity, which might then induce an increase in the dopaminergic periglomerular neurons as a compensatory plastic response to the lack of centrifugal inhibition (Mundiñano et al., 2011). Our data of unchanged NA and 5-HT levels in the *recovered* group as compared to controls do not support such an explanation for increased DA neurons in the OB, however a potential cholinergic denervation, supporting centrifugal inhibition in a recent study on PD patients and MPTP-treated (Mundiñano et al., 2013), was not determined in our study.

Interestingly, DA increase was not accompanied by equivalent increases in the DA metabolites DOPAC and HVA, which explain the decrease in the turnover ratios which turned out to be significant in the case of DOPAC/DA in the *recovered* group. This discrepancy between a high DA increase and a lower increment of HVA and unchanged DOPAC in OB tissue with potential dopaminergic neurogenesis is surprising since the SN with its intrinsic dopaminergic neurons contains relatively higher levels of DA metabolites than striatum with its dopaminergic nerve endings but no intrinsic DA neurons (Pifl and Hornykiewicz, 2006). This would have led to expect a substantial increase of metabolites in the OB by an increased number of dopaminergic cell bodies. Therefore, a profound difference can be assumed between newly born dopaminergic neurons in the OB and dopaminergic neurons in the SN in terms of metabolism. For example, synaptic vesicles with vesicular monoamine transporter within newly formed dopaminergic neurons in the OB as opposed to the endogenous without it (Peter et al., 1995; Weihe et al., 2006) might explain the high level of DA relative to DA metabolites by preventing cytosolic degradation and a high resistance of these cells to toxic effects of MPTP similarly to mice overexpressing the vesicular monoamine transporter which were reported to be protected from MPTP neurotoxicity (Lohr et al., 2014). Furthermore, the OB has dramatically less DA transporter compared with striatum (Mitsumoto et al., 2005; Cockerham et al., 2016) which is consistent with the resistance of these neurons (in all species) to destruction by either 6-hydroxydopamine or MPTP. In fact, intranasal MPTP administration in rat also leaves many dopaminergic periglomerular neurons survive, whereas dopaminergic neurons of the SN were strongly affected confirming a different vulnerability to MPTP (Prediger et al., 2009). As far as potential interference of high DA levels with normal OB function is concerned the low DA turnover based on metabolite to DA ratios could indicate a defective neurotransmission of the additional dopaminergic neurons.

Out of all the neurotransmitter related substances in OB analyzed in this study, taurine had the highest tissue levels which is in agreement with a report on taurine content of eight different major rat CNS areas with the highest value in the OB which was quite similar to the present values in monkeys (Collins, 1974). A range of cortical and subcortical regions in our *Macaca fascicularis* also contained much lower taurine levels than OB (unpublished results). The significant correlations of single values of HVA with taurine calculated over all four MPTP groups and of aspartate with taurine over the *asymptomatic* and the *recovered* but not in the *control* group suggest an interaction between DA metabolism and taurine as well as excitatory activity and taurine in the neuronal network of the OB triggered by the dopaminergic neurotoxin. MPTP does not shift the levels of these parameters to significantly changed mean values in particular groups of MPTP exposed monkeys but seems to cause perturbations with parallel individual deflections in the single animals. Although correlations do not imply causation, it is interesting in this respect that intraventricular taurine increased striatal and hypothalamic DOPAC in the rat (Panula-Lehto et al., 1992), the main DA metabolite in this species, and a neuroinhibitory effect of taurine was observed in the OB with selectivity for mitral and tufted cells (Belluzzi et al., 2004) which are glutamatergic cells (Nagayama et al., 2014).

## Conclusion

Our observation of a substantial increase of DA tissue levels confirms and expands to the non-human primate a paradoxical reaction of DA in the OB in parallel with progressive nigro-striatal dopaminergic lesion. We also show that no other monoamine neurotransmitters or established and potential amino acid neurotransmitters are affected by MPTP. This first neurochemical investigation of OB in various stages after MPTP administration suggests that the effect on DA seems to be an early phenomenon in PD, not requiring profound nigrostriatal neurodegeneration or the appearance of PD motor features. Furthermore, this study supports the accumulating evidence that neurotransmitters play a crucial role in determining survival and differentiation of newly generated neurons in the OB.

## Author Contributions

CP and JB designed the work. CP, HR, NLR, CC, JAO and JB did the acquisition, analysis, or interpretation of data for the work. All the authors drafted the work and revised it critically for important intellectual content approved the final version to be published.

## Conflict of Interest Statement

The authors declare that the research was conducted in the absence of any commercial or financial relationships that could be construed as a potential conflict of interest.
